# Bidirectional Sling Technique with Biopsy Forceps for Inferior Vena Cava Filter Retrieval

**DOI:** 10.3400/avd.hdi.20-00097

**Published:** 2021-09-25

**Authors:** Hitoshi Anzai, Satoru Takaesu, Tomoyuki Yaguchi, Takayuki Shimizu, Tatsunori Noto, Yoshinori Nagashima, Naohiko Nemoto

**Affiliations:** 1 Department of Cardiology, SUBARU Health Insurance Ota Memorial Hospital, Ota, Gunma, Japan

**Keywords:** inferior vena cava filter retrieval, bidirectional sling technique, biopsy forceps

## Abstract

Although the importance of the retrieval of an optional inferior vena cava filter (o-IVCF) has gained attention because of the awareness of a high complication rate with long indwelling time, the o-IVCF retrieval rate remains low. The advanced retrieval technique of o-IVCF may increase the retrieval rate, which in turn diminishes future adverse events. Through two cases, we describe how to perform the novel approach “bidirectional sling technique with biopsy forceps.” This technique will improve the retrieval rate in patients following the failure by conventional retrieval technique.

## Introduction

Optional infeior venacava filter (o-IVCF) development has resulted in a remarkable increase in IVCF implantation.^1)^ In recent years, several studies have raised questions regarding the safety of o-IVCF.^2)^ In August 2010, the Food and Drug Administration (FDA) announced the incidents that may have been related to the o-IVCF remaining in the body long after the risk of pulmonary embolism (PE) had subsided and encouraged physicians to consider o-IVCF retrieval when no longer indicated. Nonetheless, the device retrieval rates remain low.^3)^

In reality, to increase the device retrieval rate is more complicated than we expected, since o-IVCFs with extended implantation times present a challenge to retrieval, where conventional techniques frequently fail. If we want to maintain the technical success of o-IVCF retrieval, advanced techniques should be developed, especially in patients with longer o-IVCF indwelling time.^4)^ Thus far, several advanced techniques have been published in the literature, including sling technique, balloon dilatation technique, dissection technique with biopsy forceps, and excimer laser sheath.^5)^ However, in Japan, we do not apply excimer laser sheath, which is available overseas, to strip severely embedded IVCF hook or struts from the caval wall.

In this article, two cases that required a novel advanced technique with the use of “bidirectional sling technique with biopsy forceps” were presented for the removal of an o-IVCF.

## Case Reports

### Case 1

A 50-year-old female developed proximal deep venous thrombosis (DVT). She was waiting for a uterine corpus cancer operation. She was referred to our hospital and underwent o-IVCF (Günther–Tulip: Cook Medical Inc, Bloomington, IN, USA) implantation in suprarenal position, followed by anticoagulation therapy. After 3 months, she visited our outpatient clinic for follow-up. We planned the o-IVCF retrieval with a 4 month indwelling period since the operation was successful and she was able to take anticoagulants safely.

**Figure 1:**
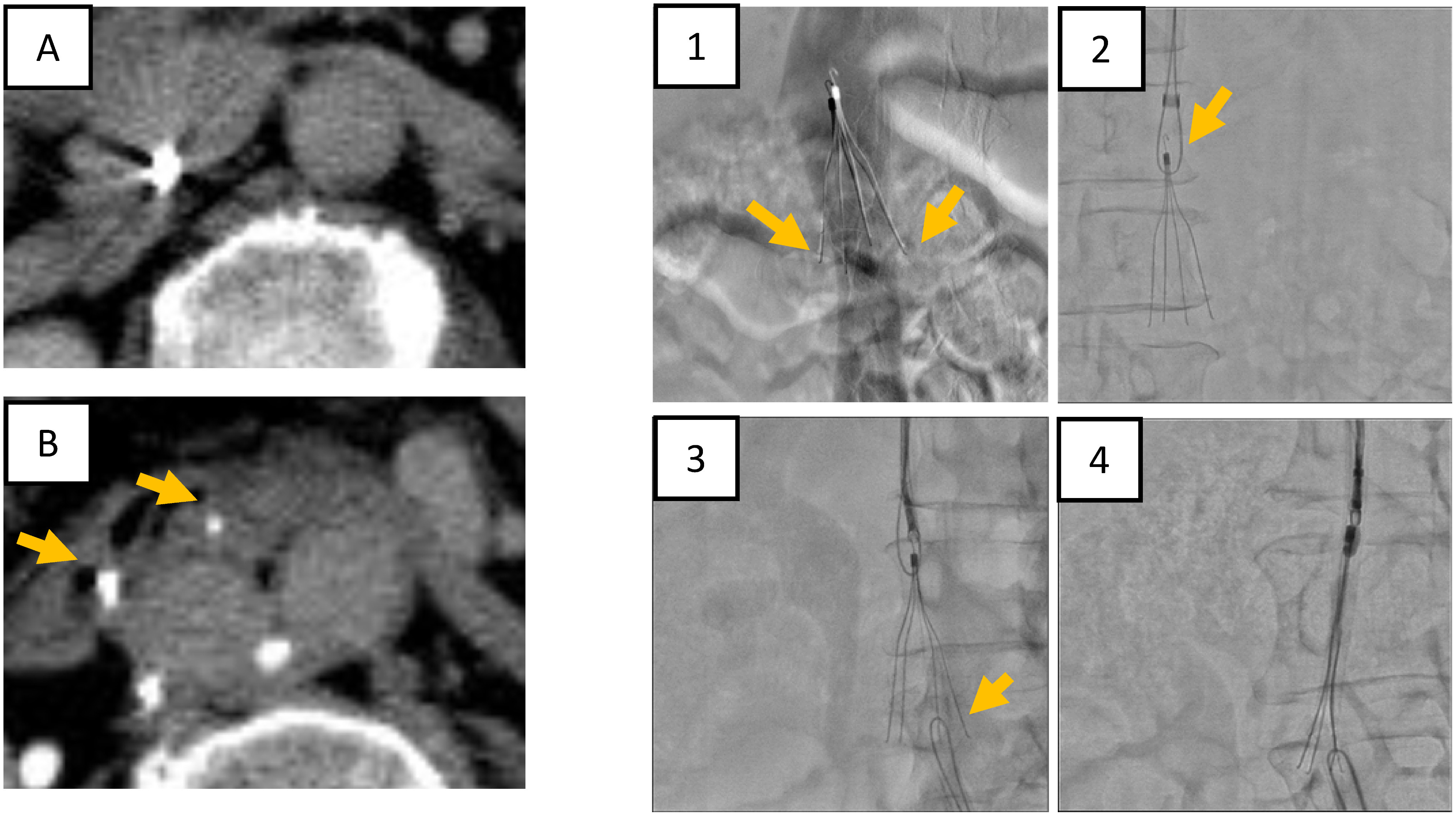
Fig. 1 Günther–Tulip optional inferior vena cava filter (o-IVCF) retrieval following 4 months of implantation. (**A**, **B**) Computed tomography indicates that the hook is probably embedded in the caval wall. Two of four legs are out of the vessel (arrows). (**1**) Initial venogram also demonstrates that some legs look like they are out of the vessel (arrows). At first, we attempt to catch the hook by the conventional snare system through the right internal jugular vein. However, it is hard to catch the hook presumably because of the embedded hook. (**2**) The conventional system is exchanged to the 11 Fr retrieval sheath within a 14 Fr sheath, and the sling technique with a 0.035 guidewire is established with the use of a Trilobe snare (EnSnare) (arrow). We are successful in grabbing the hook with a 7 Fr endomyocardial biopsy forceps, but we are not able to introduce the hook into the sheath. (**3**) We make another loop around a leg with a 0.035 guidewire and a 4 Fr JL1 diagnostic catheter (arrow) through the right femoral vein. We establish the bidirectional sling technique. The 11 Fr retrieval sheath is exchanged to a Byrd dilator sheath (inner sheath outer size, 13.6 Fr) within a 14 Fr sheath, and the hook is grabbed with the biopsy forceps and the sling is established again. (**4**) We strongly but gradually advance the Byrd sheath while pulling the 0.035 guidewire from both sides to keep the same IVCF position. The Byrd sheath slowly moves down, and eventually, we are successful in withdrawing the o-IVCF out of the right internal jugular vein sheath. The final venography has no vessel injury.

Computed tomography (CT) showed the hook was attached to the caval wall. Two of four leg struts obviously out of the vessel (**Figs. 1A** and **1B**). The initial venogram demonstrated several legs were out of the vessel (**Fig. 1**(**1**)). An 11 Fr sheath (A Günther–Tulip filter retrieval kit, Cook Medical Inc., Bloomington, IN, USA) within a 14 Fr sheath (35 cm, Medikit Co., Tokyo, Japan) was inserted in the right internal jugular vein (IJV). The first attempt using a conventional snare failed to catch the hook since the hook was embedded. Then, a 0.035-inch guidewire (Terumo, Tokyo, Japan) was dropped down under the o-IVCF. Another 0.035 guidewire was passed through separate interstices (ideally on the opposite side), exchanging to a 12/20 mm Trilobe snare (EnSnare: Merit Medical, South Jordan, UT, USA). The first 0.035 guidewire was caught by the EnSnare below the o-IVCF and was pulled up and withdrawn out of the sheath in the right IJV; the sling technique was established (**Fig. 1**(**2**)). We attempted to introduce the embedded hook into the 11 Fr sheath. Although the hook was successful in grabbing with a 7 Fr endomyocardial biopsy forceps (Cordis, Santa Clara, CA, USA), we were not able to introduce the hook into the sheath. Then, another loop was created around one of the leg struts with a 0.035 guidewire from the right femoral vein (FV) supported by a 4 Fr JL1 diagnostic catheter; the “bidirectional sling technique” was established (**Fig. 1**(**3**)). The 11 Fr sheath was exchanged to a mechanical sheath dedicated for pacemaker lead extraction made of polypropylene (a Byrd dilator sheath, outer size of the inner sheath; 13.6 Fr, Cook Medical Inc, Bloomington, IN, USA). This sheath was so rigid that it could provide adequate power to peel the adhesion tissue surrounding the o-IVCF. The same procedure was performed to establish the sling technique with the biopsy forceps again. We strongly but gradually advanced the Byrd sheath while pulling the 0.035 guidewires from both sides to keep the IVCF position at the same level, which was supposed to enhance the traction/countertraction power and to prevent IVC dissection and intussusception. The Byrd sheath was slowly advanced, and finally, the IVCF was successfully removed (**Fig. 1**(**4**)). The final venography did not show any vessel injury.

### Case 2

A 70-year-old female had hemodialysis introduced 3 years before. One year later, she had severe cholecystitis because of a common bile duct stone. While hospitalized, she developed DVT and had an o-IVCF (OptEase: Cordis, Warren, NJ, USA) deployed in the inferior vena cava. Although since then she had been on anticoagulants and in an uneventful course, she was referred to our hospital for o-IVCF retrieval with 2 year indwelling time. The principal reason for o-IVCF removal was her desire to stop taking anticoagulation.

**Figure 2:**
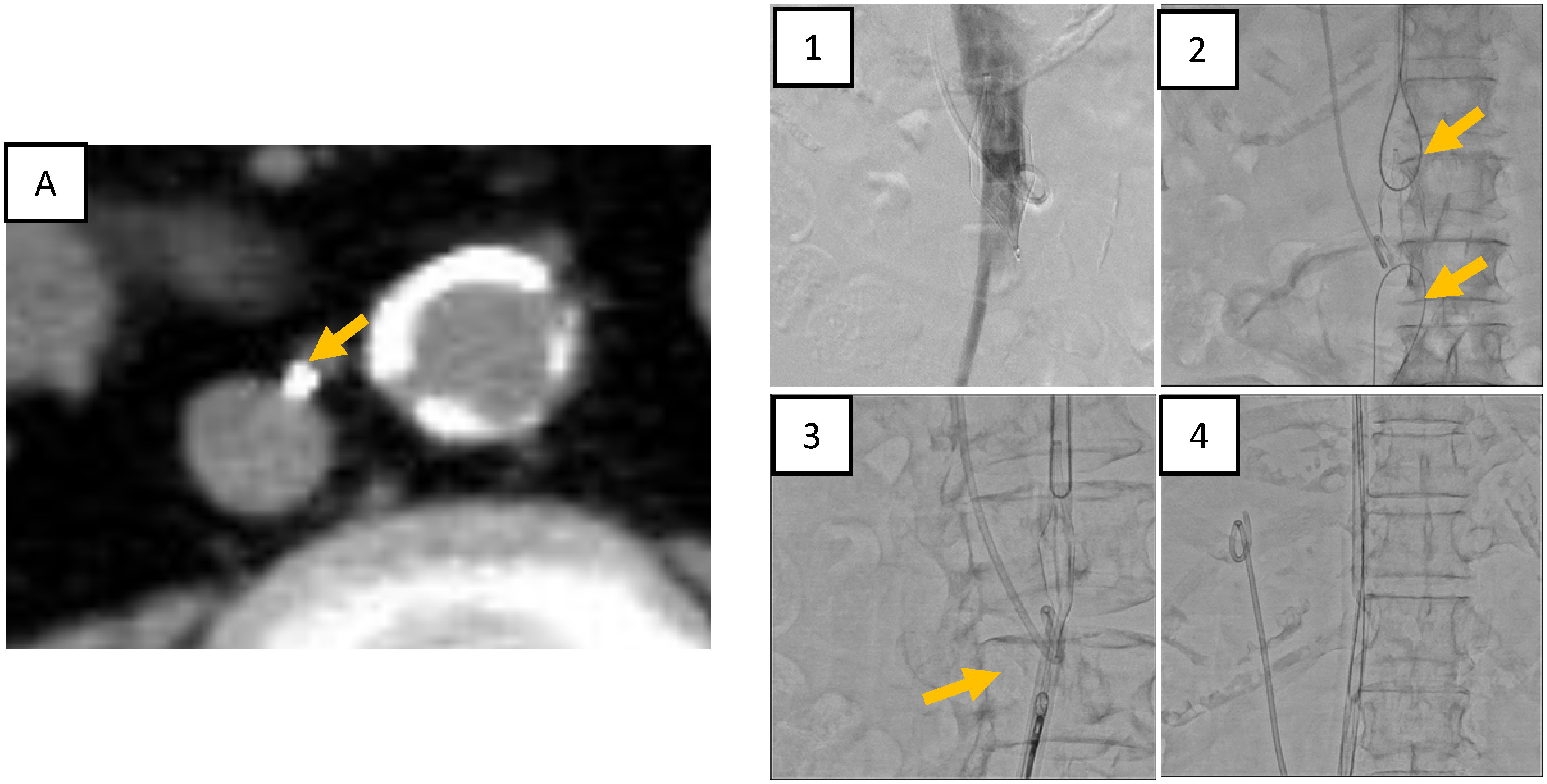
Fig. 2 OptEase optional inferior vena cava filter (o-IVCF) retrieval following 2 years of implantation. (**A**) Computed tomography shows the bottom of IVCF with hook is probably embedded in the caval wall (arrows). (**1**) The initial venography reveals IVCF struts are deeply embedded into the caval wall. A Byrd dilator sheath (inner sheath outer size, 13.6 Fr) within a 14 Fr sheath is inserted via the right femoral vein. The sling technique is established around the bottom hook with a 0.035 guidewire and a 4 Fr JL1 diagnostic catheter. Another Byrd sheath within a 14 Fr sheath is inserted via the right jugular vein, and another loop is made around the top of the IVCF. (**2**) Then, we establish the bidirectional sling technique (arrows). (**3**) We are successful in grabbing the embedded bottom hook with a 7 Fr endomyocardial biopsy forceps (arrow). We advance the Byrd dilator sheath strongly but gradually from the femoral vein while pulling the 0.035 guidewire from both sides to keep the same IVCF position. When we feel a strong resistance advancing the Byrd sheath from the femoral vein, we then advance the Byrd dilator sheath from the jugular vein. (**4**) Two Byrd sheaths clung together, and then, the IVCF is withdrawn from the femoral vein. The final venography has no vessel injury.

CT showed that the top of o-IVCF did not attach to the caval wall. Nevertheless, all six body struts were tenting against the caval wall and the bottom of the o-IVCF with the hook probably embedded in the caval wall (**Fig. 2A**). We placed a Byrd sheath within a 14 Fr sheath from both the right FV and the right IJV. The initial venography from the femoral sheath showed IVCF body struts were probably deeply embedded into the caval wall (**Fig. 2**(**1**)). At first, we created a loop around the bottom hook with a 0.035 guidewire supported by a 4 Fr JL1 diagnostic catheter. The 0.035 guidewire was caught by an EnSnare, withdrawing the guidewire out of the sheath, and the sling technique was established. Another loop was created around the top of the o-IVCF in the same manner via the Byrd sheath from the right IJV. The bidirectional sling technique was established (**Fig. 2**(**2**)). We also successfully grabbed the embedded bottom hook with a 7 Fr endomyocardial biopsy forceps and introduced the hook into the Byrd sheath. We advanced strongly but gradually the Byrd sheath from the FV while pulling the 0.035 guidewire from both sides to keep the o-IVCF position at the same level (**Fig. 2**(**3**)). We advanced the Byrd sheath from either side; when we felt a strong resistance while advancing the Byrd sheath from the FV, we started to advance the Byrd sheath from the IJV and vice versa. When the two Byrd dilator sheaths clung together and there was a loss of resistance, the o-IVCF was successfully withdrawn and came out of the sheath in the FV (**Fig. 2**(**4**)). The final venography did not reveal any vessel injury.

## Discussion

The FDA disclosed in 2010 that it had received greater than 900 reports involving problems with retrievable filters, some of which resulted in adverse clinical outcomes. Hence, the FDA issued the safety communications that physicians ongoing care of patients with o-IVCF must consider device retrieval when no longer needed.^6)^ Following the statements of the FDA, the importance of the o-IVCF retrieval has gained general attention, and the o-IVCF retrieval rate has increased over the last period, but it remains suboptimal.^7)^ There may be several causes of filter left despite initial plans for retrieval: death, need for ongoing PE protection, loss to follow-up, and physician oversight. Furthermore, failed retrieval is one of the principal reasons.^8)^

To address the difficulty in removing o-IVCF and achieve a more increasing overall retrieval rate, we need advanced techniques beyond a conventional one. To date, several advanced techniques have been suggested in the literature.^5)^ The commonly encountered reasons for retrieval failure are as follows: ① the tilting of IVCF, leading to a problem in engaging the apex or hook into a sheath with a standard snare catheter, and ② the incorporation of IVCF struts with the caval wall.^5)^

To solve problem ①, we sometimes need to apply biopsy forceps, which can grasp the hook or apex of tilted IVCF and introduce it into a sheath. Additionally, we can dissect the tissues around the embedded apex of IVCF by biopsy forceps and release the apex of IVCF from the caval wall. How to manipulate biopsy forceps is one of the keys to successful retrieval. We use a 7 Fr endomyocardial biopsy forceps with a manual bending of 1–1.5 cm of the tip for making it to a certain direction. It can be inserted in an 11 Fr retrieval sheath or a Byrd dilator sheath (inner sheath outer size, 13.6 Fr) with two 0.035 guidewires. We can control the direction of the tip of biopsy forceps and confirm grasping the apex of IVCF with the biopsy forceps under multiple fluoroscopic projections. As for problem ②, the introduction of the sling technique, making a loop around the apex of IVCF with 0.035 guidewire, can provide sufficient traction force. Based on our experience, most of the patients following retrieval failure with the conventional technique can be addressed with the sling technique with or without biopsy forceps. However, since excessive adhesion around the hook or struts develops with time in some patients, much stronger traction force is needed in some cases. The possible advantages of the “bidirectional sling technique” are strengthening of traction/countertraction force and prevention of potential complications like caval wall dissection and intussusception.^9)^

Ross et al.^10)^ reported the same concept using the bidirectional approach and emphasized the advantage of making a loop snare from both sides by maximizing axial stability. They also stressed second loop snare was able to strip the leg anchors from the caval wall. In this article, we described the benefit of keeping an IVCF position at the same level during pulling an IVCF in terms of the prevention of dissection and intussusception. This complication would happen by dragging adhered struts along the axis of the vessel when an IVCF is strongly pulled even before it was not fully introduced into a sheath. We presume that the risk of these complications would be obviated by keeping the level of IVCF position. Moreover, we believe this could provide more chance of retrieval success by increasing traction/countertraction force by pulling IVCF from both sides. Also, Ross et al.^10)^ described this technique as a last resort because the presence of multiple wires and points of attachment creates an environment that risks entanglement of wires. However, we would like to propose that this technique is easily added in the middle of a procedure any time when we feel more powerful traction force is needed.

We used a Byrd sheath made of polypropylene, dedicated for pacemaker lead extraction. The property of this sheath is rigid and can be rotated while being advanced. Under the current circumstance that excimer laser sheath is not allowed to apply for IVC filter retrieve in Japan, the Byrd sheath can be an alternative device for dissecting severely embedded struts of IVCF against the caval wall.

While the development of advanced retrieval techniques has greatly improved the retrieval rates, the use of advanced techniques is associated with a remarkably higher complication rate.^9)^ Thus, the use of the advanced retrieval technique should be weighed against the benefit of filter retrieval on a patient-to-patient basis.

## Conclusion

We present two cases with successful o-IVCF retrieval with the “bidirectional sling technique.” The use of the “bidirectional sling technique” is a feasible approach in addition to advanced techniques with a single sling technique and biopsy forceps. We believe that this technique will improve the retrieval rate of o-IVCF in patients with failure with the use of the conventional retrieval technique.
